# Cargo crowding contributes to sorting stringency in COPII vesicles

**DOI:** 10.1083/jcb.201806038

**Published:** 2020-05-13

**Authors:** Natalia Gomez-Navarro, Alejandro Melero, Xiao-Han Li, Jérôme Boulanger, Wanda Kukulski, Elizabeth A. Miller

**Affiliations:** 1 Medical Research Council Laboratory of Molecular Biology, Cambridge, UK

## Abstract

Accurate maintenance of organelle identity in the secretory pathway relies on retention and retrieval of resident proteins. In the endoplasmic reticulum (ER), secretory proteins are packaged into COPII vesicles that largely exclude ER residents and misfolded proteins by mechanisms that remain unresolved. Here we combined biochemistry and genetics with correlative light and electron microscopy (CLEM) to explore how selectivity is achieved. Our data suggest that vesicle occupancy contributes to ER retention: in the absence of abundant cargo, nonspecific bulk flow increases. We demonstrate that ER leakage is influenced by vesicle size and cargo occupancy: overexpressing an inert cargo protein or reducing vesicle size restores sorting stringency. We propose that cargo recruitment into vesicles creates a crowded lumen that drives selectivity. Retention of ER residents thus derives in part from the biophysical process of cargo enrichment into a constrained spherical membrane-bound carrier.

## Teaser

Combining correlative light and electron microscopy with yeast genetics and biochemistry, Gomez-Navarro, Melero, and colleagues show that cargo recruitment into a constrained COPII vesicle restricts bulk flow, thereby contributing to sorting stringency and ER quality control.

## Introduction

Protein trafficking within the eukaryotic secretory pathway occurs via cargo-bearing vesicles that shuttle proteins and lipids from one compartment to another. Cytosolic coat proteins drive vesicle formation by deforming the membrane of the donor organelle into small carriers and selecting cargo proteins for incorporation into the carrier vesicles (for reviews see Bonifacino and Lippincott-Schwartz, 2003; Dancourt and Barlowe, 2010; Geva and Schuldiner, 2014). The first step taken by nascent secretory proteins is packaging into coat protein II (COPII)–coated vesicles that bud from the ER for delivery to the Golgi (Barlowe et al., 1994; Gürkan et al., 2006; Lee et al., 2004). The COPII coat assembles on the ER membrane in two layers. The inner cargo- and lipid-bound layer comprises the small GTPase, Sar1, and the cargo adaptor complex, Sec23/Sec24. This inner coat in turn recruits an outer coat of heterotetrameric Sec13/Sec31, which forms rod-like structures that can self-assemble into a polyhedral cage that is thought to contribute to vesicle architecture (Fath et al., 2007; Noble et al., 2013; Zanetti et al., 2013). In addition to the five core COPII coat proteins, regulatory components control vesicle formation at discrete ER exit sites (ERES). Sec16 is one example of an accessory protein that is thought to define sites for COPII recruitment and assist in coat assembly (Supek et al., 2002; Kung et al., 2012).

ER exit can be highly selective: in some cell types and in in vitro reconstitution experiments, properly folded secretory proteins are enriched in COPII vesicles, and ER resident proteins are largely excluded (Barlowe et al., 1994). Indeed, despite high concentrations of ER resident proteins (Macer and Koch, 1988), secretion of ER chaperones and folding intermediates is minimal, although in part this effect is driven by efficient signal-mediated retrieval of escaped ER residents (Munro and Pelham, 1987). Cargo enrichment into COPII vesicles is mediated by direct interaction between ER export signals and Sec24, which contains multiple independent cargo-binding sites (Miller et al., 2003; Mossessova et al., 2003; Mancias and Goldberg, 2007, 2008). Protein sorting is also facilitated by cargo receptors that bridge the interaction between cargo and coat proteins (Geva and Schuldiner, 2014). In addition to signal-mediated trafficking, proteins can also move within the secretory pathway by bulk flow, whereby proteins are not enriched in vesicles but are stochastically captured at their prevailing concentrations as part of the bulk fluid or membrane (Martínez-Menárguez et al., 1999; Wieland et al., 1987; Polishchuk et al., 2003; Thor et al., 2009).

One of the consequences of cargo enrichment in vesicles is the potential for macromolecular crowding to create steric pressure that can oppose the action of the coat machinery (Derganc et al., 2013; Stachowiak et al., 2013). Evidence for such crowding effects comes from experiments in yeast, where secretion of a particularly abundant family of secretory proteins, the glycosylphosphatidylinositol-anchored proteins (GPI-APs), can be modulated genetically. GPI-APs are packaged into COPII vesicles via interaction with the p24 family of proteins (Castillon et al., 2011). Deletion of any of the four major yeast p24 proteins (Emp24, Erv25, Erp1, and Erp2) results in viability in the absence of Sec13, known as a bypass of sec-thirteen (*bst*) phenotype (Elrod-Erickson and Kaiser, 1996; Belden and Barlowe, 2001; D’Arcangelo et al., 2015). One model for the *bst* phenotype is that enrichment of GPI-APs at ERES creates a local domain that is resistant to membrane deformation (Copic et al., 2012; D’Arcangelo et al., 2015). This rigid membrane requires the COPII coat to do extra work to enforce curvature, which is contributed in part by Sec13. Thus, in p24 mutants, where GPI-AP enrichment is reduced, the absence of Sec13 is tolerated because less force is required to overcome the membrane bending energy at an ERES.

In addition to the *bst* phenotype, p24 mutants also have defective retention of ER resident and misfolded proteins, and a constitutive activation of the unfolded protein response (UPR). The molecular basis for these phenotypes remains poorly understood, including how the different cellular outcomes relate to each other. These various phenotypes have led to models for p24 proteins functioning in ER retention by modulating the timing of vesicle release (Kaiser, 2000), or by displacing nonspecific cargo (Kaiser, 2000; Ma et al., 2017). Here we aimed to better understand the consequences of p24 deletion, focusing on two aspects: membrane bending by Sec31 alone, and selectivity of ER export. We examined vesicle morphology in situ in the absence of Sec13, and probed mechanisms by which the p24 proteins might act as a selectivity filter. We find that in the absence of Sec13, COPII-associated membranes become large and pleiomorphic. In contrast, ER leakage in the *emp24Δ* mutant derives from alterations in cargo occupancy rather than vesicle morphology or specific p24 function. We propose that inclusion of p24 proteins and other abundant cargoes in COPII vesicles generates macromolecular crowding that disfavors capture of ER residents.

## Results

**Figure 1.**
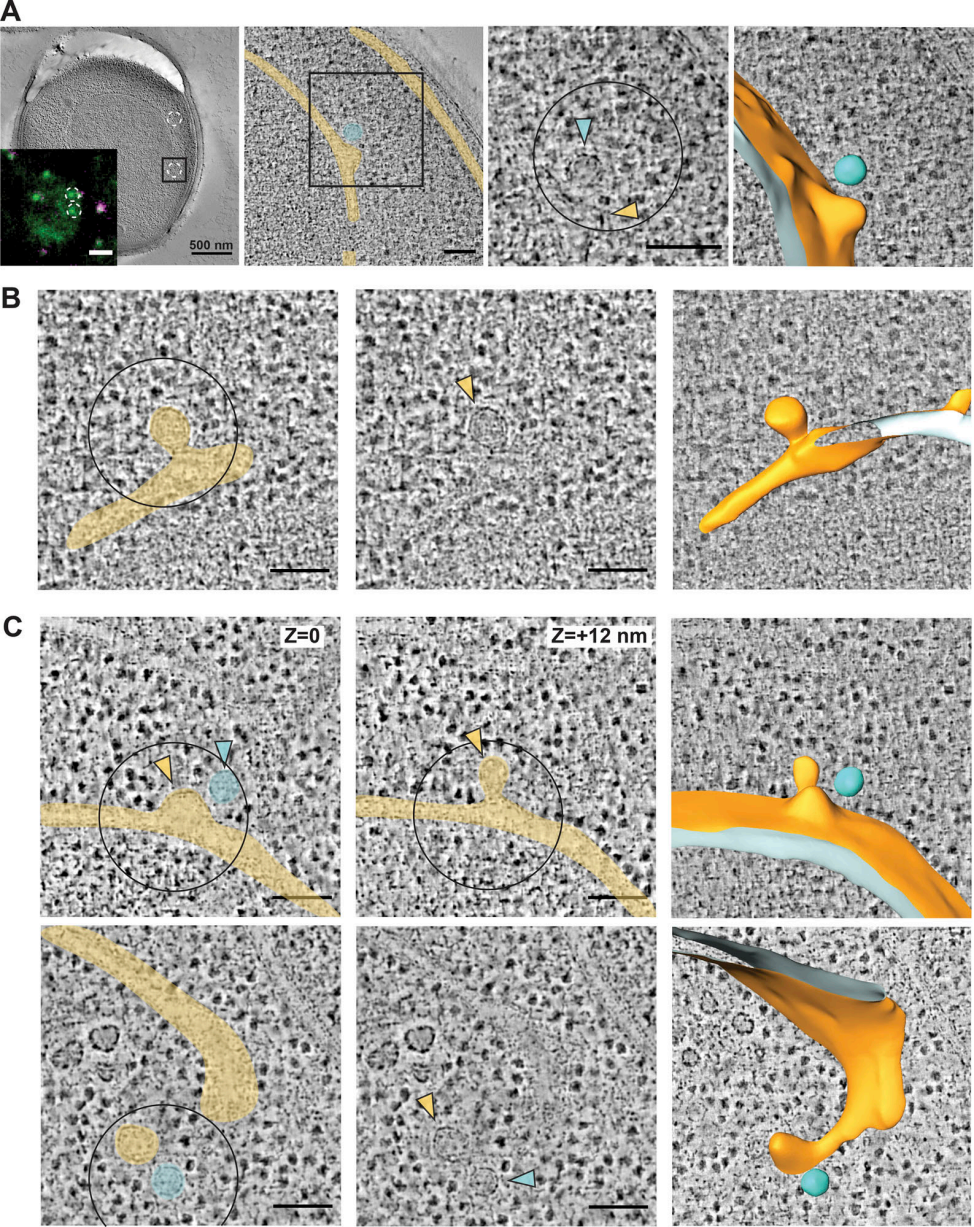
**3D ultrastructure of COPII budding events and free vesicles by CLEM.** **(A)** Resin-embedded yeast cell containing ERES correlated to Sec24-sfGFP signal. Left panel, inset: two Sec24-sfGFP foci (green) are highlighted. Fiducial markers used for correlation are visible in green and magenta. Left panel, a virtual slice of a low magnification tomogram used for correlation with fluorescent image of the same cell (inset). Middle-left panel is a virtual slice of a high magnification tomogram on one of the two Sec24-sfGFP marked ERES, highlighted by a 500 x 500 nm square. Nuclear envelope and ER cisterna are false colored in yellow, and a free vesicle is highlighted in cyan. Middle-right panel is a zoom-in on the same virtual slice as the previous panel. The center of the 250-nm-diameter circle marks the predicted position of the GFP signal centroid. Colored arrowheads mark a nascent COPII bud (yellow) and a free vesicle (cyan), represented in a segmentation model on the farthest right panel. **(B)** A virtual tomographic slice showing a correlated Sec24-sfGFP ERES. Left panel, ER is false-colored in yellow. Central panel, arrowhead marks the emerging bud. Right panel is a segmentation model of the budding event. **(C)** Upper panels: two virtual tomographic slices of a single Sec16-sfGFP correlated spot: different z positions of the same x,y position are shown, revealing a multibudded ERES with two buds (yellow) and a free vesicle (cyan). Lower panels are a virtual tomographic slice showing a correlated Sec16-sfGFP ERES. Left panel, ER is false colored in yellow. Central panel, arrowheads mark an emerging bud (yellow) and a free vesicle (cyan). Right panel is a segmentation model of the budding event. Scale bars: A, EM, 500 nm; inset, 1 µm; all other panels, 100 nm.

**Figure S1.**
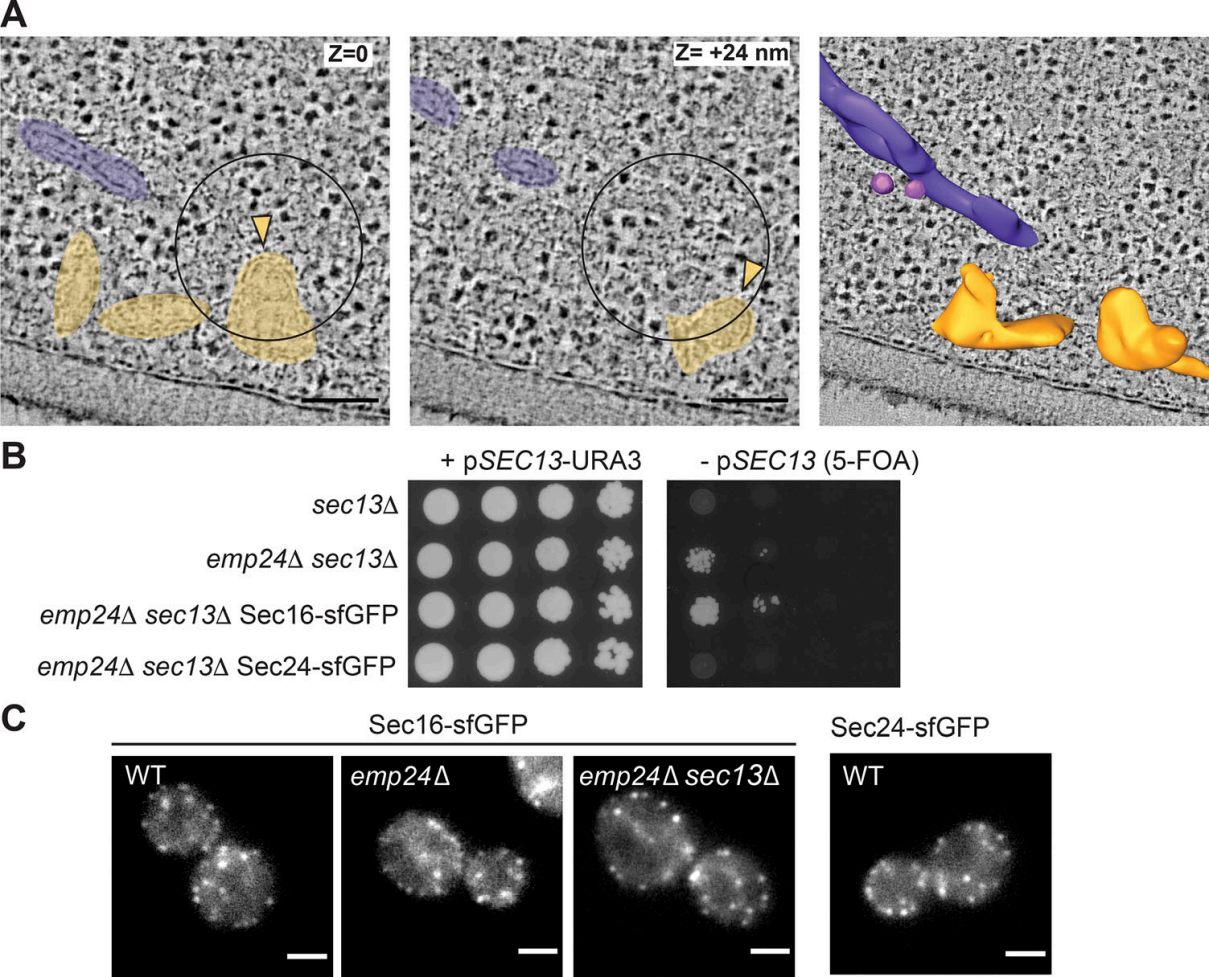
**CLEM tomography of COPII-associated membranes.** **(A)** Two virtual tomography slices of a single Sec24-sfGFP positive ERES. Different heights within the correlation are marked (z), showing a multibudded ERES with two buds (yellow) and a Golgi complex cisterna (purple). Scale bars, 100 nm. **(B)** Serial dilutions of the indicated strains were spotted as serial dilutions onto media containing 5-FOA to counter select for the *SEC13-URA3* plasmid and test for viability. On standard media (left panel), all strains grew, whereas growth in the absence of *SEC13* (5-FOA; right panels) was only observed in an *emp24*Δ background. Chromosomal tagging of *SEC16* was tolerated in this background, whereas tagged *SEC24* was not viable. **(C)** Fluorescence microscopy of the indicated strains expressing *SEC16-sfGFP* and a *SEC24-sfGFP* WT strain. Scale bars, 2 µm.

To understand the effects of cargo enrichment on vesicle formation and ER retention, we first sought to visualize the ultrastructure of ERES with high spatial resolution. This approach allows us to measure how membranes and vesicles in cells change in the absence of specific cargo and coat components. Sites of COPII vesicle formation can be localized in cells expressing fluorescently tagged COPII subunits (Watson et al., 2006; Okamoto et al., 2012). However, membrane morphology falls below the diffraction limit such that detailed structural information can only be obtained by EM (Orci et al., 1991; Zeuschner et al., 2006). To exploit the advantages of fluorescent protein localization and the resolution of electron tomography, we used a correlative light and EM (CLEM) approach (Kukulski et al., 2011). We tagged Sec24 at its chromosomal locus by integrating superfolder GFP (sfGFP) at the C terminus (Sec24-sfGFP) and subjected cells to high pressure freezing, freeze substitution, and resin embedding. Thick sections (∼300 nm) were collected on EM grids, labeled with fluorescent fiducial markers, and imaged by fluorescence microscopy to identify GFP-positive ERES (Fig. 1 A, inset). Sections were subsequently imaged by electron tomography at both low (Fig. 1 A, left panel) and high magnification (Fig. 1 A, center and right panels). Fiducial markers permit the precise spatial correlation of the Sec24-sfGFP signal within the electron tomograms, allowing visualization of the underlying membranes in this region (Fig. 1 A, right panels). We found a range of membrane morphologies at Sec24-positive ERES, including flat ER membranes, budding events with a nascent vesicle still continuous with the ER, ERES with multiple buds, and free vesicles released from ER membranes (Fig. 1, A and B; and Fig. S1 A).

We also introduced sfGFP at the chromosomal locus of *SEC16* of WT cells, revealing similar ERES as Sec24-sfGFP (Fig. S1 C). By CLEM we observed similar membrane morphologies associated with both Sec16-sfGFP (Fig. 1 C) and Sec24-sfGFP (Fig. 1, A and B; and Fig. S1 A). Together, Sec24-sfGFP and Sec16-sfGFP strains yielded a combined dataset of 127 electron tomographic reconstructions of sites of COPII vesicle formation.

### ERES are pleiomorphic in the absence of Sec13

To visualize COPII budding events in the absence of Sec13, we attempted to chromosomally GFP-tag Sec24 in the *sec13Δ emp24Δ* strain, but were unsuccessful, suggesting that a tag on Sec24 is not well tolerated in this background (Fig. S1 B). Instead, Sec16-sfGFP in this mutant background showed viability similar to that of the *sec13*Δ *emp24*Δ parental strain (Fig. S1 B), and ERES similar to those of Sec24-sfGFP in WT cells (Fig. S1 C).

**Figure 2.**
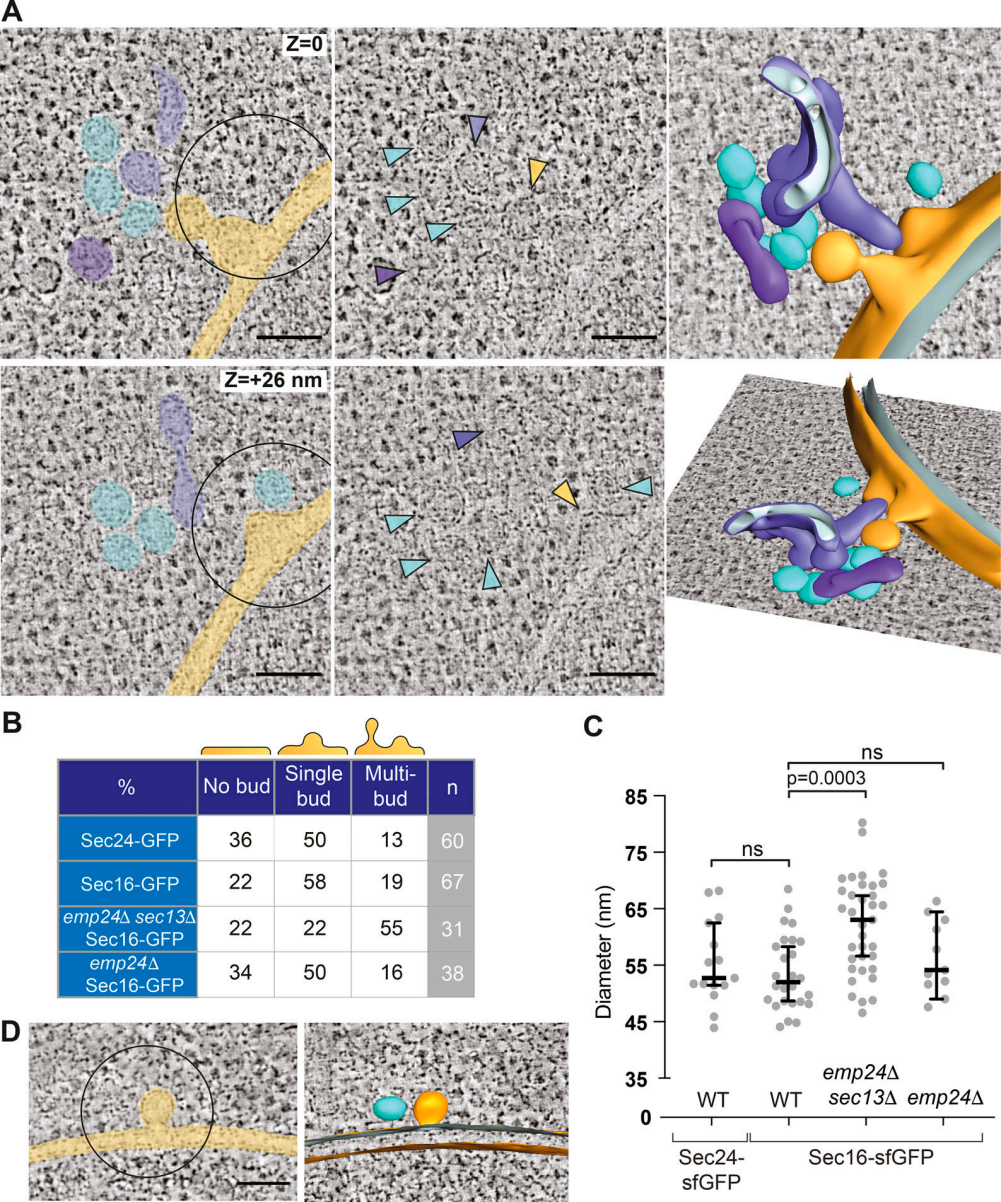
**Deletion of *SEC13* results in pleomorphic membranes at ERES.** **(A)** *SEC16-sfGFP-*positive ERES localized by CLEM in an *emp24*Δ *sec13*Δ cell. Upper and lower panels are different virtual slices from the same tomogram, representing different z-positions. Two buds form at the nuclear envelope (yellow) with six free vesicles (cyan) and two undefined tubular compartments (purple) in close proximity. In the central panels, colored arrowheads mark the same membrane structures. Right panels are two side views of a segmentation model of the ERES. **(B)** Table of ERES ultrastructure categories (percentages from total number [n] of correlated spots per yeast strain). **(C)** Plot of maximum diameter (nm) of vesicles for the different strains indicated; *n* = 15 for Sec24-sfGFP, *n* = 26 for Sec16-sfGFP, *n* = 35 for *emp24*Δ *sec13*Δ, *n* = 11 for *emp24*Δ. Bars correspond to median value and 95% confidence interval. Statistical test was a one-way ANOVA with Tukey’s correction for multiple comparisons; ns, not significant. **(D)** A *SEC16-sfGFP-*positive ERES localized by CLEM in an *emp24*Δ *SEC16-sfGFP* cell. A bud emerges from the nuclear envelope (yellow) with a free vesicle by the side (cyan). 3D segmentation model on the right. Scale bars, 100 nm.

**Figure S2.**
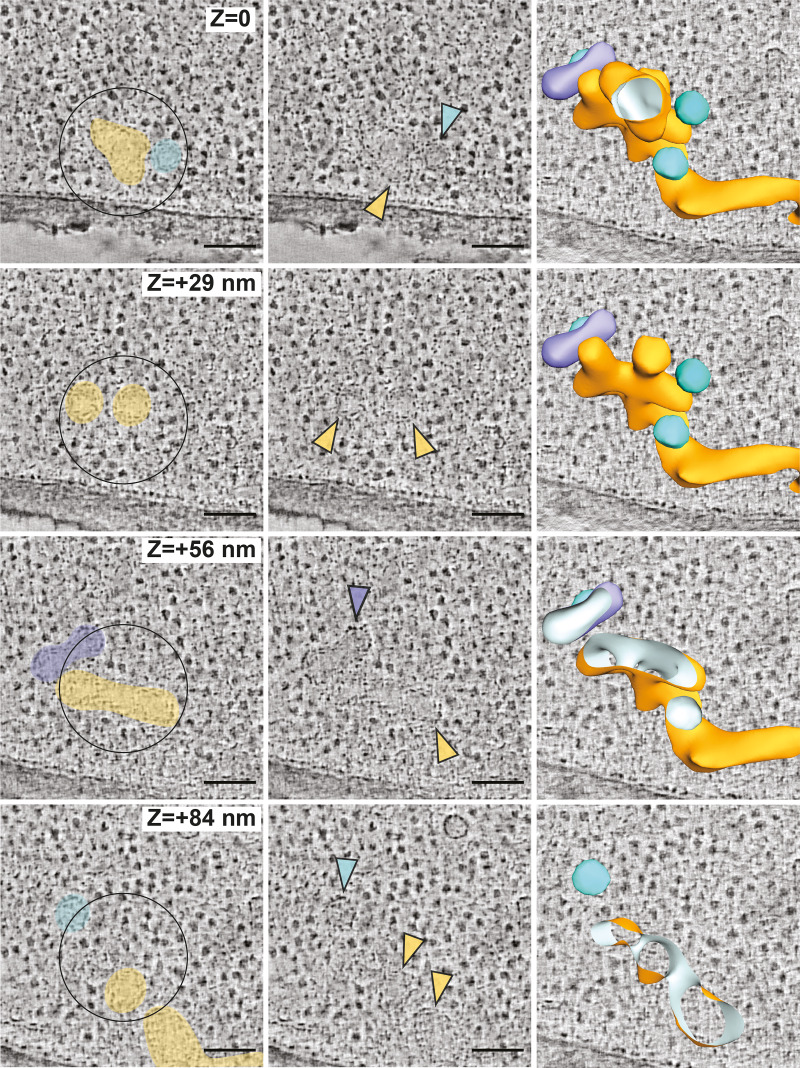
**Virtual tomogram of an *emp24Δ sec13Δ* ERES.** **(A)** Virtual tomography slices through the 3D volume (z) of an *emp24*Δ *sec13*Δ cell expressing *SEC16-sfGFP*. Left panels show colored ER (yellow), vesicles (cyan), and an unidentified tubular compartment (purple). Central panels show the same structures highlighted with colored arrowheads. Left panels are cut-throughs of a segmentation model of the 3D ultrastructure of membranes at the ERES. Scale bars, 100 nm.

Having validated that similar structures can be observed with Sec16-sfGFP as with Sec24-sfGFP in WT cells, we next applied CLEM to *sec13*Δ *emp24*Δ *SEC16-sfGFP* cells. In the absence of Sec13, the N-terminal β-propeller region of Sec31 is thought to remain intact and capable of assembling into a polymeric cage (Fath et al., 2007; Stagg et al., 2006; Copic et al., 2012). We anticipated that the flexible hinge region of Sec31 exposed by loss of Sec13 should yield a less rigid structure with reduced membrane bending capacity, thereby generating enlarged COPII buds and vesicles. We acquired 31 tomograms at Sec16-sfGFP–correlated ERES in *sec13Δ emp24Δ* cells (Fig. 2 A and Fig. S2). The corresponding membrane ultrastructures showed broadly similar characteristics as those in WT cells, encompassing flat ER membranes, and budded and multibudded structures. However, the sites in *sec13Δ emp24Δ* cells appeared more pleomorphic, with numerous buds and vesicles (Fig. 2 A and Fig. S2).

To quantify the different morphologies of ERES, we designated three broad categories of structures: flat ER membranes without an obvious bud (no bud), ER membranes with a single budded structure still attached (single bud), and structures with multiple curved membrane structures (multibud; Fig. 2 B). Comparison of the classes from different strains revealed no obvious difference between WT ERES marked with either Sec24-sfGFP or Sec16-sfGFP. Most ERES in both WT strains showed a single budding event, while multibudded structures were less frequent. In contrast, the *sec13Δ emp24Δ* strain had fewer single bud profiles and many more multibudded membranes (Fig. 2 B). As a second measure of membrane morphology, we used a quantitative segmentation analysis to measure the sizes of free vesicles (Machado et al., 2019). Maximum diameters of vesicles ranged from 45 nm to 65 nm, with a median of 52 nm (Fig. 2 C). Vesicles in *sec13Δ emp24Δ* cells showed a broader size distribution than in WT cells, with a median diameter 18% larger than WT (Fig. 2 C). We note that the smallest WT vesicle diameters of 45–50 nm were also found in *sec13*Δ *emp24*Δ cells, suggesting that Sec13 is not absolutely required to achieve high curvature (Fig. 2 C).

The large pleiomorphic structures of COPII-associated membranes in the *sec13Δ emp24Δ* strain suggest an obvious mechanism of lax ER retention, one of the shared phenotypes of the p24 mutants. When larger structures bud from the ER, bulk flow capture would increase, thereby stochastically packaging more ER residents and misfolded nascent proteins. This could overwhelm Golgi–ER retrieval mechanisms mediated by saturable receptors, such as the His-Asp-Glu-Leu (HDEL) receptor, Erd2, that recycles ER residents back to the ER (Semenza et al., 1990). Receptor saturation would result in leakage of ER residents to the cell surface (Schuldiner et al., 2005), which can be readily monitored by detection of an extracellular pool of the lumenal HSP70, Kar2, and is a known phenotype of the p24 mutants. We examined the morphology of Sec16-sfGFP–associated ERES in the *emp24Δ* single mutant to determine if large membrane structures might explain ER retention defects in single mutant cells. However, we observed largely normal membranes: In 38 tomograms of Sec16-sfGFP–positive regions (Fig. 2 D), most had the simple ERES morphology observed in WT cells comprising a single nascent bud (Fig. 2 B). Multibudded structures were less common in the *emp24Δ* single mutant than in the *sec13Δ emp24Δ* double mutant, and were observed at a similar frequency as in WT cells. Furthermore, WT cells and the *emp24Δ* single mutant showed similar size distributions and median diameters of free vesicles (45–65 nm, median of 54 nm; Fig. 2 C).

The normal membrane structures observed in the *emp24Δ* single mutant cells suggest that vesicle size alterations cannot explain the lax ER retention phenotype of p24 mutants (Elrod-Erickson and Kaiser, 1996). Instead, we hypothesized that reduced cargo occupancy in the mutant vesicles might create space that would allow for stochastic capture of ER resident proteins. The CLEM approach does not allow us to visualize cargo proteins within vesicles, so we turned to biochemical analyses to test this hypothesis. We first sought to rule out various indirect effects of a p24 deletion that might result in ER retention defects: (1) chaperone induction via the UPR could saturate ER retrieval (Belden and Barlowe, 2001); (2) ER resident chaperones could be exported from the ER while bound to misfolded clients, which might be expelled from the ER under stress (Satpute-Krishnan et al., 2014); (3) increased diffusional mobility of proteins in the ER lumen could promote access to ERES (Lai et al., 2010); and (4) altered Golgi–ER retrieval could increase secretion.

### ER retention defects in p24 mutant strains are not explained by ER stress-induced effects

**Figure 3.**
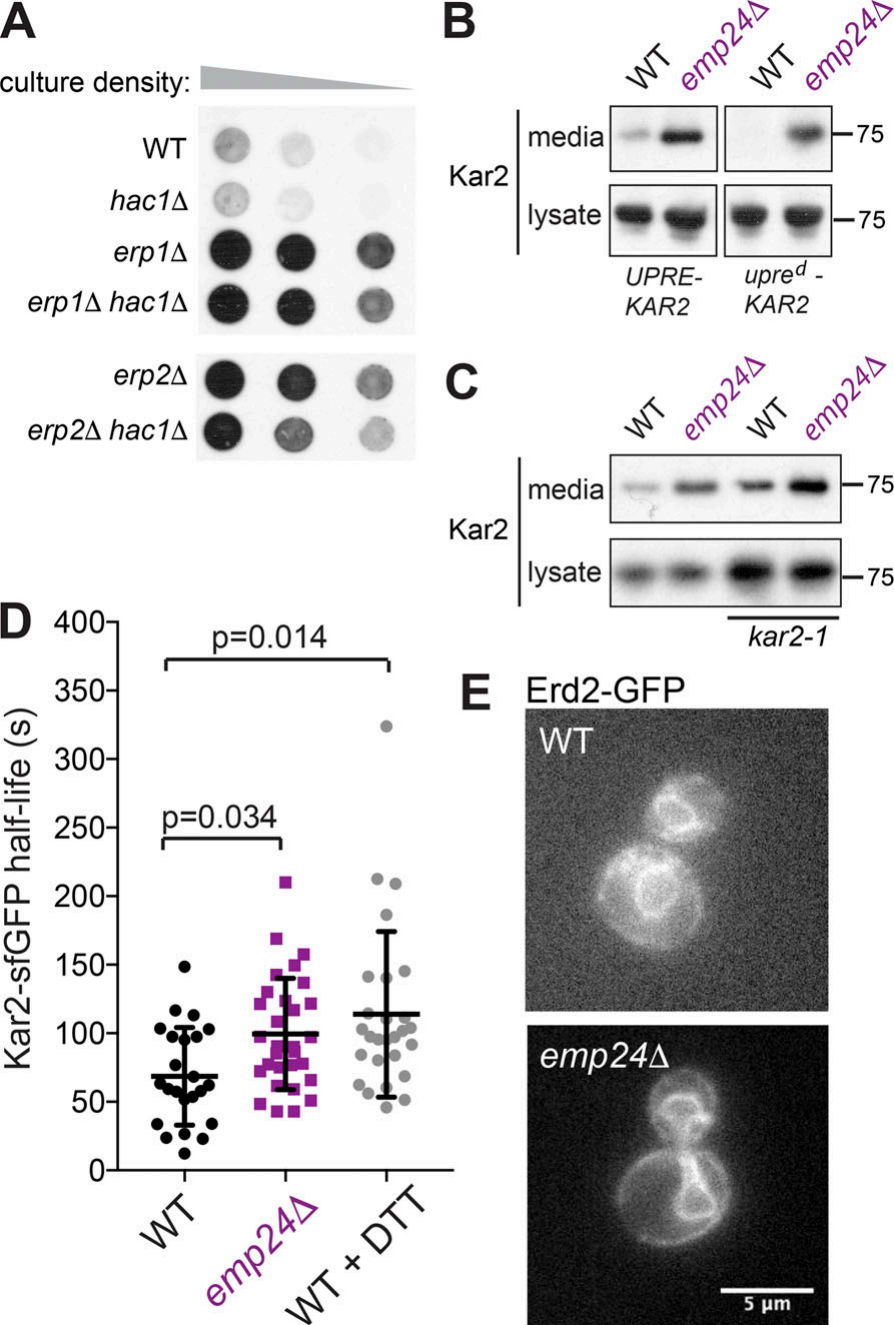
**Kar2 secretion is not due to UPR, retrieval failure, or changes in ER lumenal mobility.** **(A)** Serial dilutions of the indicated yeast strains were overlaid with nitrocellulose, and secreted Kar2 detected with Kar2-specific antibodies. **(B and C)** Kar2 was detected in intracellular (lysate) and secreted (media) fractions by SDS-PAGE and immunoblotting with anti-Kar2 antibodies. **(D)** Mobility of Kar2-sfGFP was measured by FLIP. Kar2 half-life in individual cells was measured in the indicated strains. WT + DTT cells were treated with 5 mM DTT for 1 h. The graph shows the mean and the error bars represent SD; *n* = 23 (WT); *n* = 30 (*emp24Δ*); *n* = 27 (WT + DTT). Statistical test was a one-way ANOVA with Dunnett’s correction for multiple comparisons. **(E)** Fluorescence microscopy of WT and *emp24Δ* cells expressing Erd2-GFP revealed ER localization in both strains.

We first tested UPR effects by deleting Hac1, the transcription factor responsible for UPR activation, in two relatively mild p24 mutants, *erp1Δ* and *erp2Δ*. In these backgrounds, robust secretion of the abundant ER lumenal HSP70, Kar2, was still observed in a colony immunoblot assay even when the UPR is abrogated (Fig. 3 A). Since deletion of *EMP24* is inviable in the absence of the UPR (Copic et al., 2009), we sought an independent means to test UPR dependence in the *emp24Δ* strain. Abrogation of the UPR element within the *KAR2* promoter yields a strain, *upre^d^*-*KAR2,* in which *KAR2* expression is uncoupled from ER stress (Hsu et al., 2012). We deleted *EMP24* in this background and found no reduction in Kar2 secretion, confirming that Kar2 leakage in p24 mutant strains continues even when protein levels are not modulated by UPR induction (Fig. 3 B). We next tested whether Kar2 secretion in the *emp24Δ* strain results from its ER expulsion in complex with misfolded clients, which might be triggered by ER stress (Satpute-Krishnan et al., 2014). We deleted *EMP24* in a *kar2-1* mutant strain, in which client binding is abrogated (Kabani et al., 2003). This strain has a constitutive UPR and hence up-regulates Kar2 (Fig. 3 C, lysate). This condition leads to elevated Kar2 secretion in a WT background, but *EMP24* deletion further enhanced Kar2 secretion, suggesting p24-driven release is independent of client interaction (Fig. 3 C).

To address the diffusional mobility of Kar2 within the ER lumen, we used fluorescence loss in photobleaching (FLIP) to measure the half-life of Kar2-sfGFP. In this assay, a region of the cell containing cortical ER is continuously photobleached, and the loss of fluorescence in regions outside the photobleached region quantified. This gives a measure of the diffusional mobility of Kar2-sfGFP, which has previously been demonstrated to decrease significantly under conditions of ER stress (Lajoie et al., 2012). We similarly observed an increase in Kar2-sfGFP half-life (i.e., decreased mobility) upon treatment with DTT (Fig. 3 D). The *emp24Δ* mutant also showed reduced Kar2-sfGFP diffusion (Fig. 3 D), consistent with the known constitutive UPR activation in this strain. This observation that the ER lumen is not more diffusive in *emp24Δ* cells argues against the hypothesis that elevated export of ER resident proteins results from enhanced access to ERES that is normally restrained by limiting the diffusion of these proteins. Finally, we sought to address whether Golgi–ER retrieval of escaped Kar2 by the HDEL receptor Erd2 was impaired in the *emp24Δ* strain. We examined the localization of Erd2-GFP, which normally localizes to the ER due to rapid Golgi–ER traffic (Schuldiner et al., 2005). A similar localization was observed in the *emp24Δ* mutant, suggesting that retrieval is functional in these ER-retention mutants (Fig. 3 E).

### ER-retention mutants show higher rates of bulk flow

**Figure 4.**
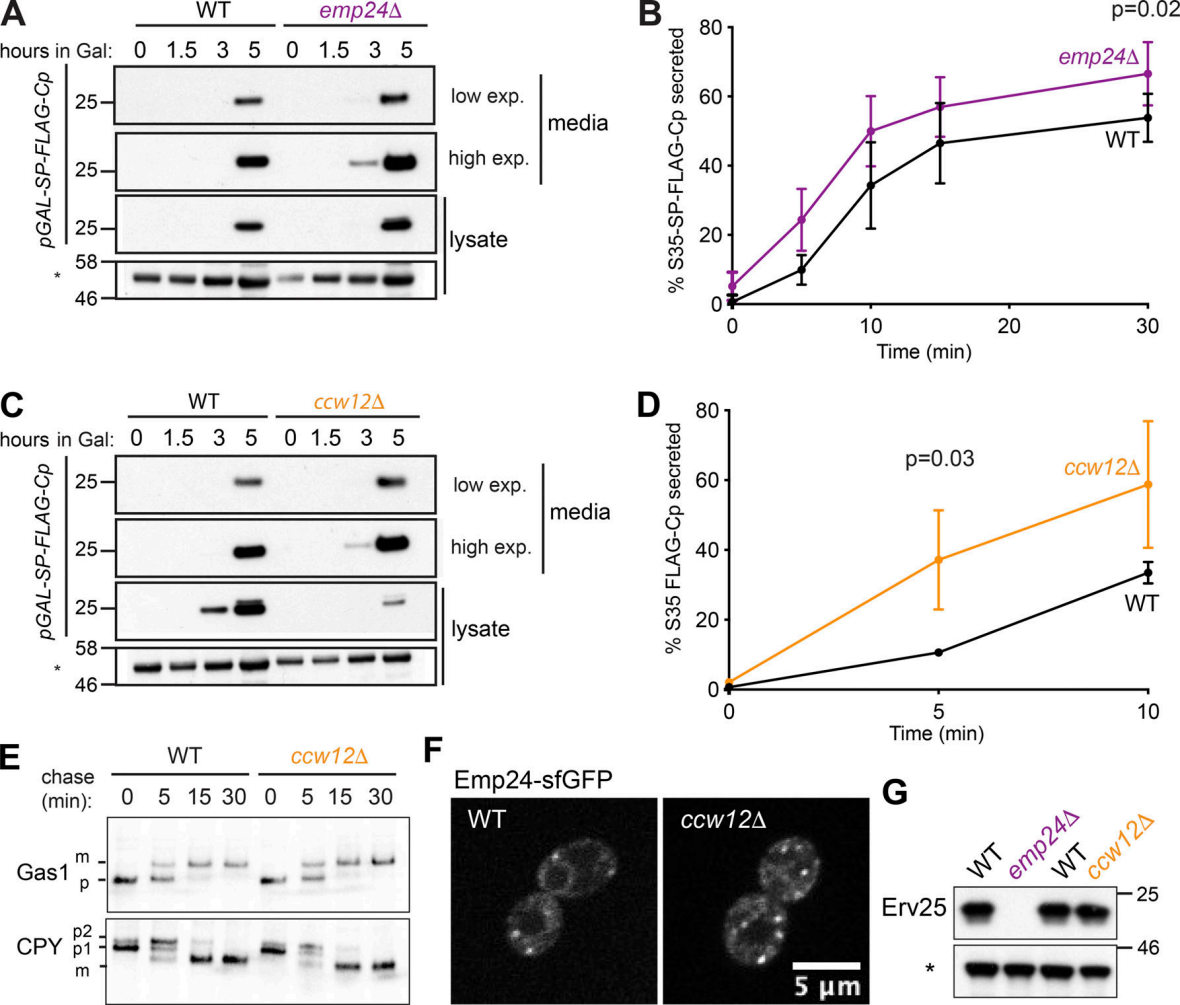
**Elevated bulk flow stems from decreased cargo crowding.** **(A)** WT and *emp24Δ* strains expressing *GAL_pr_-SP-FLAG-Cp* were induced with 0.02% galactose, and separated into intracellular (lysate) and extracellular (media) fractions. Cp was detected by SDS-PAGE and anti-FLAG immunoblot. **(B)** FLAG-Cp was immunoprecipitated from media and lysate fractions of [^35^S]methionine-labeled cells at the indicated times, analyzed by SDS-PAGE, and detected by autoradiography. The percentage of secreted FLAG-Cp is plotted for the indicated times. Error bars depict SD; *n* = 6. **(C)** As described in A. **(D)** As described in B. Error bars depict SD; *n* = 3. Statistical tests were *t* tests. **(E)** Gas1 and CPY maturation were examined in WT and *ccw12Δ* strains by pulse chase with [^35^S]methionine. Gas1 and CPY were immunoprecipitated from lysates at the indicated times and detected by SDS-PAGE and autoradiography. **(F)** Fluorescence microscopy of WT and *ccw12Δ* cells expressing Emp24-sfGFP revealed similar localization of Emp24. **(G)** Steady-state levels of Erv25 in the indicated strains were measured from whole cell lysates by immunoblotting using an Erv25-specific antibody. A nonspecific band labeled with an asterisk is shown as loading control in A, C, and G.

Having ruled out various indirect effects of p24 deletion on Kar2 secretion, we aimed to test the model that increased bulk flow could explain leakage of ER residents in these and other mutant strains. We first measured secretion of an inert marker, the C-terminal domain of the Semliki Forest virus capsid protein (Cp). This small protease has been previously used as fluid-phase marker as it folds rapidly in a chaperone-independent manner, does not undergo covalent modifications, and is unlikely to possess binding signals for cargo receptors (Thor et al., 2009). We generated a yeast version of this marker that used an Ost1 signal peptide (SP), followed by a FLAG epitope, and the Cp domain (SP-FLAG-Cp). We note that the published mammalian construct uses an HA epitope, which reveals a Tyr-Pro-Tyr ER export signal following signal peptide cleavage that might function as a cryptic ER export signal by interaction with Erv29 (Yin et al., 2018). Upon galactose induction of the bulk flow marker, we observed significant secretion in WT cells, consistent with constitutive bulk flow. In the *emp24Δ* strain, Cp secretion was elevated, suggesting bulk flow is enhanced in this background (Fig. 4 A). Quantification of Cp secretion using radioactive pulse-chase revealed an increase of ∼24% in the *emp24*Δ strain at t = 30 min (Fig. 4 B). Together, our observations are consistent with increased bulk flow rates as a cause of ER leakage in *emp24Δ* mutants. We note that this model assumes that the rate of COPII vesicle formation is unaltered when cargo abundance is reduced. This assumption is supported by in vivo imaging of ERES upon cycloheximide treatment and UPR activation (Shindiapina and Barlowe, 2010), and is in contrast to mammalian cells, where ERES abundance changes with cargo burden (Farhan et al., 2008).

**Figure S3.**
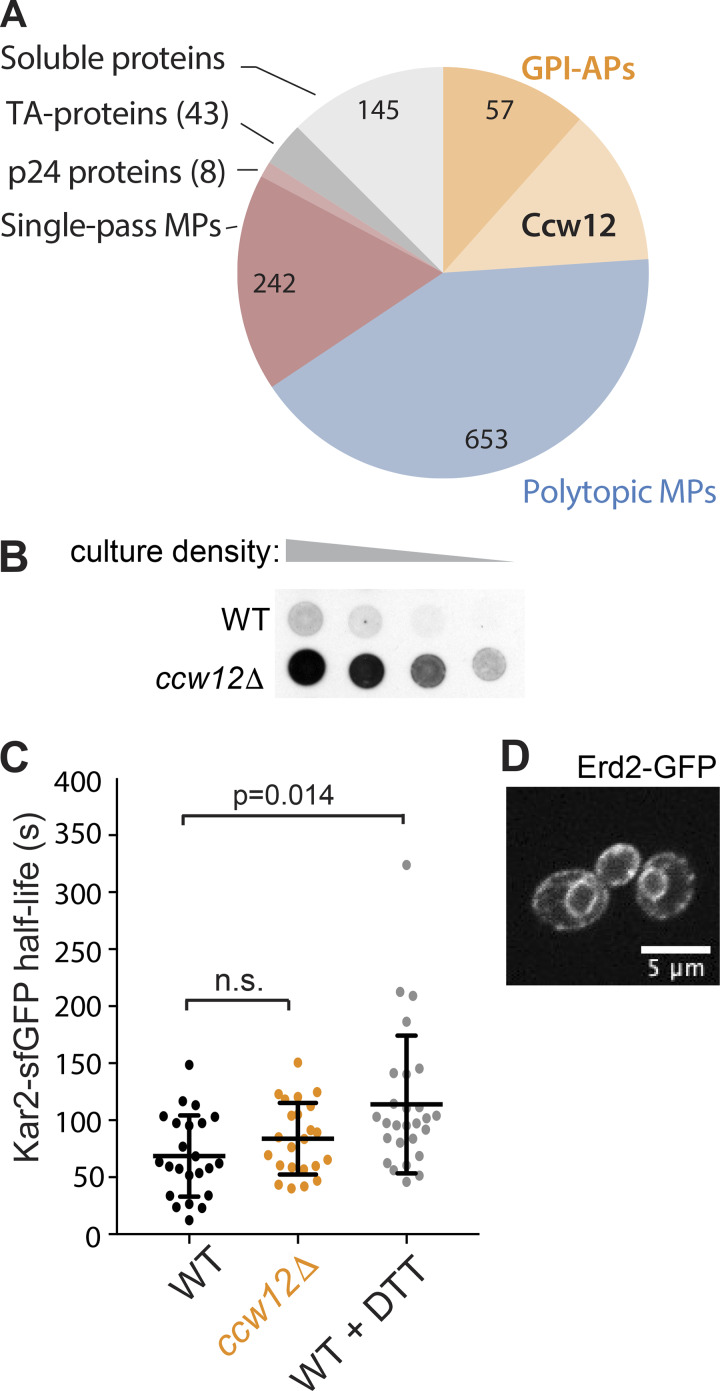
**Loss of the abundant cell wall protein, Ccw12, phenocopies an *emp24Δ* strain.** **(A)** Pie chart of GFP fluorescence of N-terminally tagged secretome proteins (Yofe et al., 2016). Cell wall proteins represent ∼25% of the GFP-secretome, with Ccw12 alone contributing ∼12% of the fluorescent signal. TA, tail-anchored; MP, membrane protein. **(B)** Serial dilutions of WT and *ccw12Δ* yeast strains were overlaid with nitrocellulose, and secreted Kar2 detected with Kar2-specific antibodies. **(C)** Mobility of Kar2-sfGFP was measured by FLIP. Half-time values, calculated as described in Fig. 3 D, of single cells are plotted for the indicated strains or WT-treated cells with 5 mM DTT for 1 h. Error bars represent SD; *n* = 23 (WT); *n* = 27 (WT + DTT); *n* = 23 (*ccw12*Δ). Statistical test was a one-way ANOVA with Dunnett’s correction for multiple comparisons. **(D)** Fluorescence microscopy of *ccw12Δ* cells expressing Erd2-GFP revealed ER localization.

We next sought to test whether enhanced bulk flow was a unique feature of the p24 mutants, or whether this model could also apply to ER retention mutants that are not integral parts of the ER-Golgi trafficking machinery. Ccw12 is a highly abundant cell wall GPI-AP, comprising ∼12% of the GFP-secretome (Ghaemmaghami et al., 2003; Caro et al., 1997; Fig. S3 A). Deletion of *CCW12* causes Kar2 secretion (Fig. S3 B; Copic et al., 2009), so we reasoned that the simple loss of this one cargo protein may mimic the p24 mutant condition by creating empty space in a vesicle to permit nonspecific capture. Indeed, secretion of FLAG-Cp was increased in a *ccw12Δ* strain (Fig. 4, C and D), consistent with increased bulk flow leakage of ER lumenal proteins. Loss of Ccw12 had no impact on Kar2 mobility (Fig. S3 C) or Erd2 localization (Fig. S3 D), and ER export rates of the vacuolar protease, Carboxypeptidase Y (CPY), and the cell wall glycoprotein, Gas1, were normal (Fig. 4 E). Normal Gas1 secretion is indicative that p24 function itself is not compromised in the *ccw12Δ* strain, but we further excluded that ER leakage might be indirectly caused by aberrant p24 function by examining recruitment of Emp24-sfGFP to ERESs, which was normal (Fig. 4 F). Moreover, deletion of *CCW12*, unlike *EMP24* deletion, did not destabilize the other main p24 protein Erv25 (Fig. 4 G). Together, the phenotypes of the *ccw12Δ* strain suggest that simple cargo occupancy can at least partially explain the decreased stringency of ER export in p24 and other mutants. We note that in vitro experiments using cycloheximide to deplete nascent secretory cargo did not result in Kar2 incorporation into vesicles (Yeung et al., 1995). However, in vitro packaging of lumenal secretory proteins is relatively inefficient (Adolf et al., 2019) such that Kar2 in a cargo-depleted vesicle population may be below the threshold of detection. We propose that enrichment of abundant cargo proteins, especially glycosylated cell wall proteins that are likely to occupy significant space, creates steric pressure within the vesicle lumen that helps exclude ER resident proteins and diminish bulk flow.

### Vesicle occupancy restrains bulk flow

**Figure 5.**
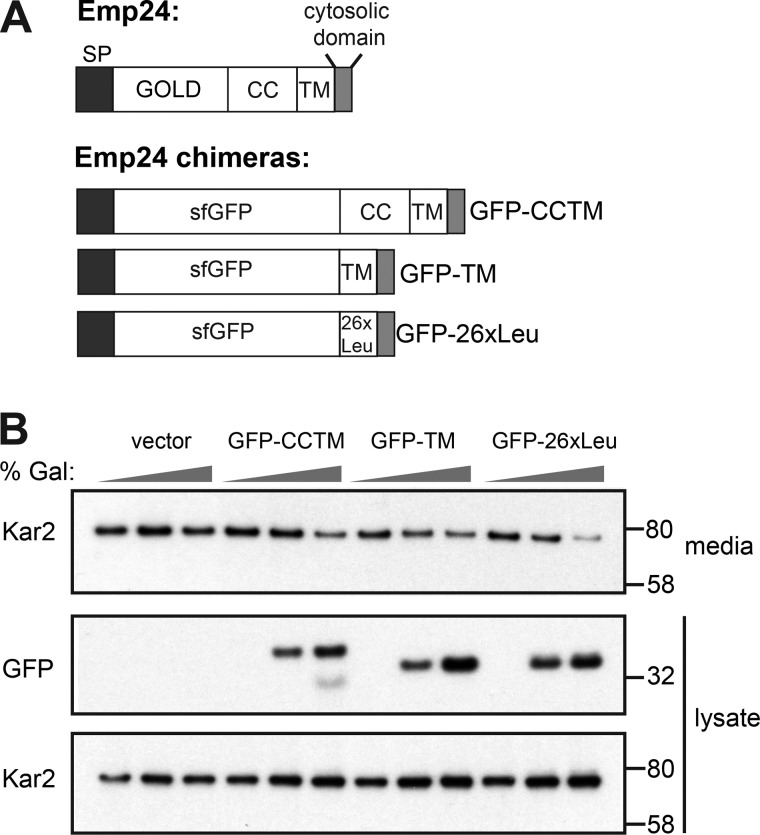
**Restoring cargo occupancy reverses ER leakage.** **(A)** Schematic of Emp24 and chimeras used in B; GOLD, Golgi dynamics domain; CC, coiled-coil; TM, transmembrane domain. **(B)** Kar2 secretion was analyzed in *emp24Δ* cells after galactose induction of the constructs indicated. Intracellular (lysate) and extracellular (media) proteins were resolved by SDS-PAGE and detected by Western blot against Kar2 and GFP.

**Figure S4.**
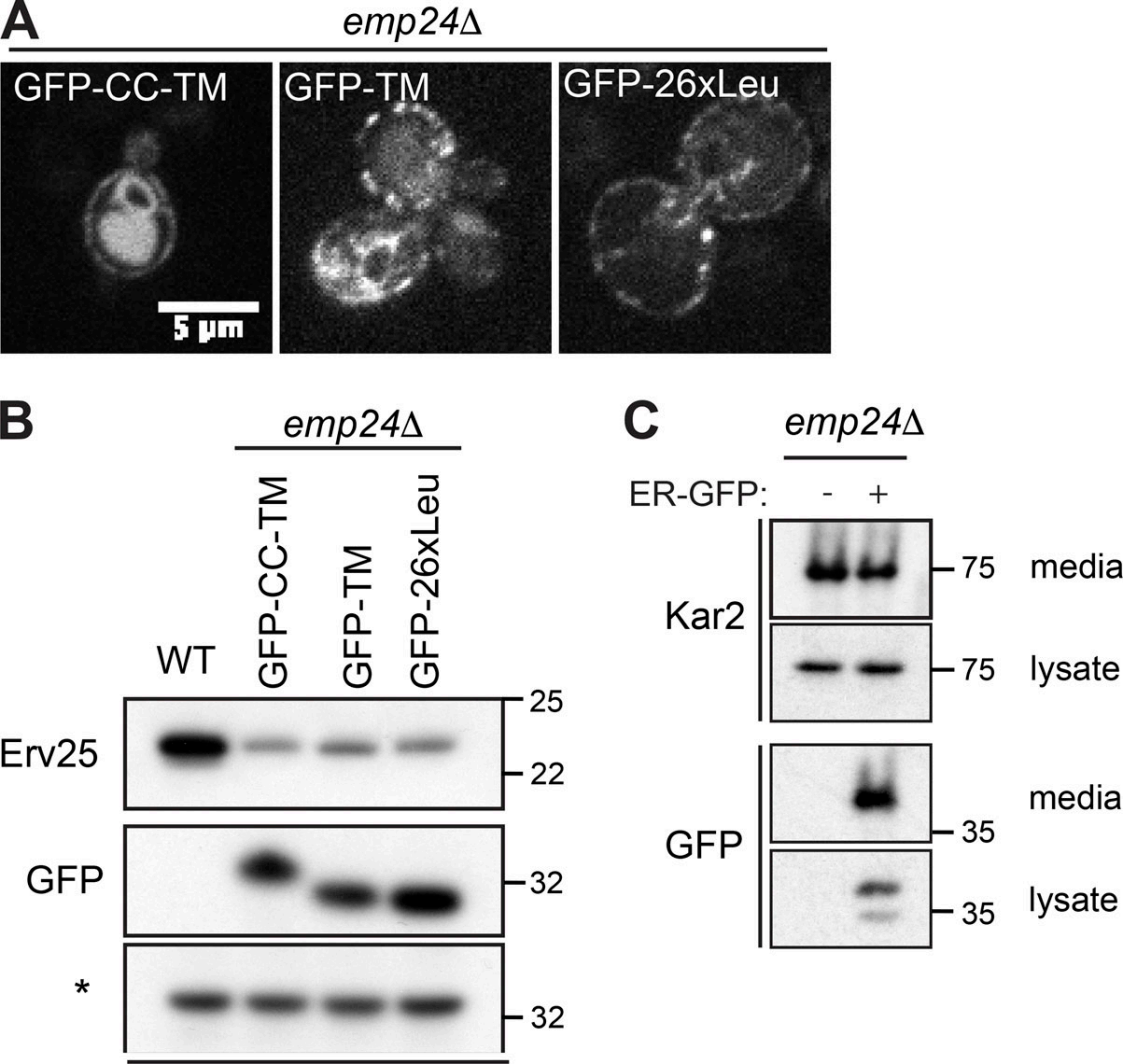
**Characterization of modular Emp24 chimeras**. **(A)** Fluorescence microscopy of *emp24Δ* cells expressing the indicated Emp24 chimeras. **(B)** Steady-state levels of Erv25 in WT cells and *emp24Δ* cells expressing the different chimeras indicated were measured from whole cell lysates by immunoblotting using an Erv25-specific antibody. Expression of chimeric proteins was detected from whole cell lysates using an antibody against GFP. A nonspecific band labeled with an asterisk is shown as loading control. **(C)** The amounts of secreted Kar2 and ER-GFP were analyzed in *emp24Δ* mutants with and without the ER-GFP plasmid. Secreted proteins in the extracellular media were concentrated using TCA; intracellular proteins were extracted with SDS. Intracellular (lysate) and extracellular (media) proteins were resolved by SDS-PAGE and detected by Western blot against Kar2 and GFP.

If Kar2 secretion is a consequence of increased bulk flow caused by increased lumenal volume available for stochastic capture, then modulating cargo occupancy should influence bulk flow. In this context, we sought to test whether p24 proteins have a specific role in modulating sorting stringency at the ER or whether local concentration of cargo directly restrains bulk flow. We tested the effects of specific p24 domains on bulk flow by using chimeric proteins that comprise a cleavable signal peptide and various substitutions of lumenal and transmembrane domains of Emp24 (Fig. 5 A). All constructs preserved the short cytosolic domain that contains the export signal responsible for capture into a vesicle. We replaced the lumenal GOLD (for Golgi dynamics) domain with sfGFP, generating the chimeric protein GFP-CC-TM (where GFP is followed by the Emp24 coiled-coil [CC] and transmembrane [TM] domains). We also deleted the short helical coiled-coil region, thought to participate in p24 oligomerization (GFP-TM), and replaced the transmembrane domain with a generic 26 Leu repeats (GFP-26xLeu). Each of these constructs was introduced into an *emp24Δ* strain, driven by the strong *GAL1* promoter. All proteins could be visualized in the ER, plasma membrane, and vacuole (Fig. S4 A) consistent with ER export and onward traffic. We monitored Kar2 secretion upon galactose induction, observing a decrease in extracellular Kar2 as the levels of the chimera proteins, serving as cargo, increased (Fig. 5 B). None of the chimeras stabilized Erv25, suggesting they are not capable of functional oligomerization (Fig. S4 B). That each chimera was capable of restoring sorting stringency suggests that simple cargo occupancy in a vesicle, rather than specific functions of the p24 proteins, is what drives selectivity. Whether organization into an array-like structure upon coat binding also contributes to this selectivity filter remains to be further explored. We note that enrichment of these chimeric cargo proteins in vesicles is essential for their reversal of ER leakage. Similar reversal was not observed when other soluble bulk flow cargoes were tested for competition (Fig. S4 C).

### Bulk flow is modulated by vesicle size

**Figure 6.**
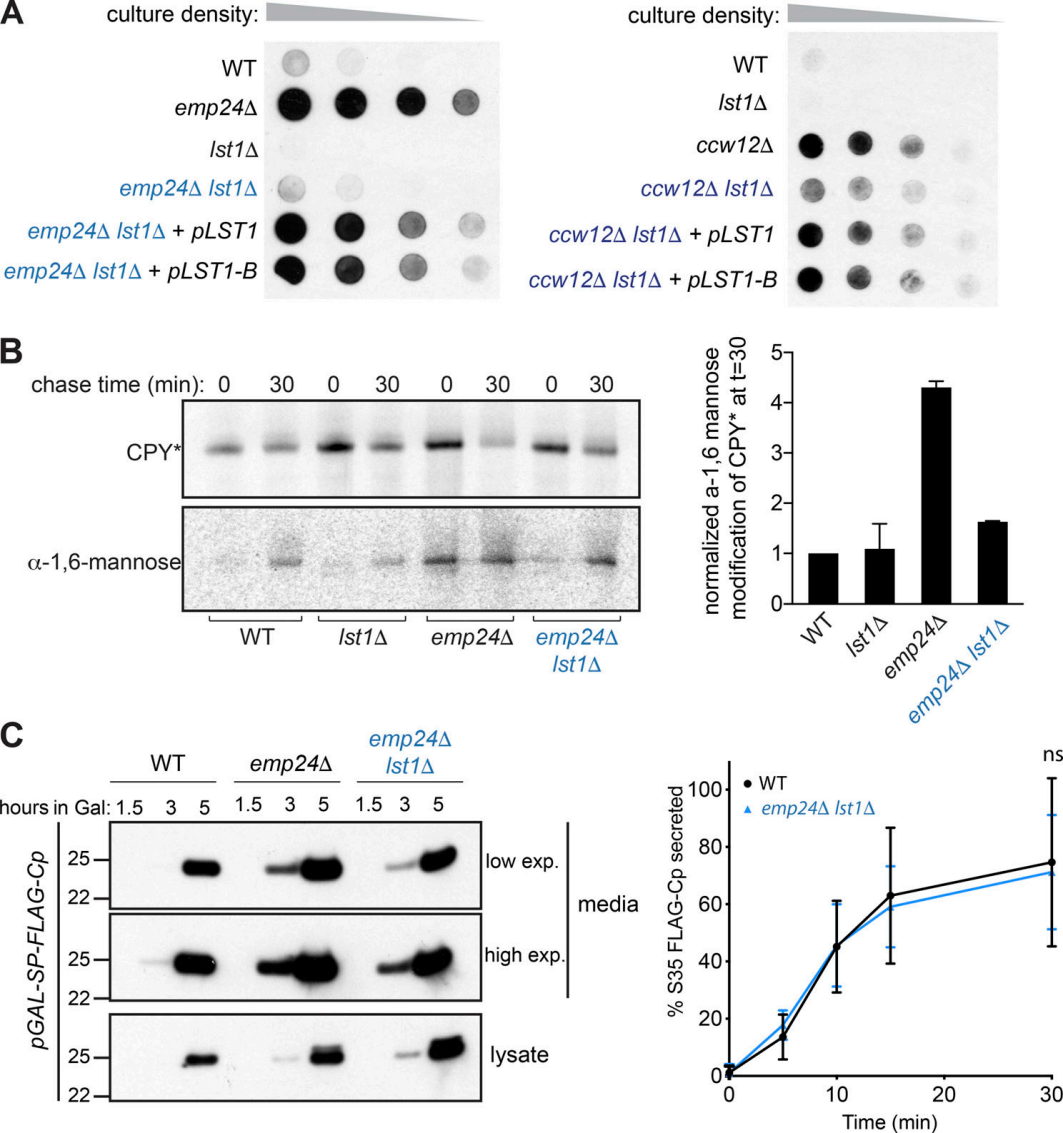
***LST1* deletion restores cargo stringency and reduces bulk flow.** **(A)** Serial dilutions of the indicated strains were spotted onto YPD plates and Kar2 secretion determined by colony immunoblot as described in Fig. 3 A. **(B)** WT, *lst1Δ, emp24Δ*, and *emp24Δ lst1Δ* cells expressing HA-CPY* were subjected to pulse chase analysis. CPY* was immunoprecipitated from lysates at the indicated times and either analyzed directly or subjected to secondary immunoprecipitation using anti-α-1,6-mannose antibodies. Immunoprecipitated proteins were resolved by SDS-PAGE and detected by autoradiography. The ratio of Golgi-modified CPY* to total CPY* relative to a WT strain at t = 30 min was determined for three independent experiments. Averages and SDs are plotted (*n* = 3). **(C)** FLAG-Cp was detected in strains indicated after induction with 0.02% Galactose as described in Fig. 4 A (left panel). Pulse-labeled proteins were immunoprecipitated from the media and lysates at the indicated times as described in Fig. 4 B. Error bars depict SD; *n* = 3 (right panel); statistical test was a *t* test.

If cargo occupancy is a main constraint on bulk flow, then vesicle size should also influence efficiency of stochastic cargo capture. Specifically, reducing vesicle size should restore a steric constraint. In yeast, COPII vesicle size is influenced by the cargo adaptor layer; vesicles formed in vitro with the Sec24 paralog Lst1/Sfb3 are ∼15% larger in diameter than those formed with Sec24 (Miller et al., 2002; Shimoni et al., 2000; Lee et al., 2002). Thus, *lst1*Δ cells should generate small Sec24-only vesicles, thereby imposing a greater crowding effect on the vesicle lumen. We examined Kar2 secretion in *emp24*Δ and *ccw12*Δ strains in the absence and presence of Lst1. Deletion of *LST1* reversed Kar2 secretion in these backgrounds, consistent with restoration of steric pressure and subsequent decrease in bulk flow leakage. This reversal was rescued by reintroduction of either *LST1* or *lst1-B*, which is mutated in the cargo-binding site (Fig. 6 A). This suggests that the effect of *LST1* deletion is related to its structural role rather than cargo capture.

Since steady-state Kar2 secretion is influenced by both anterograde and retrograde pathways, we sought to test whether the *lst1*Δ effect was specific for COPII-mediated forward traffic. We thus examined leakage of misfolded CPY, CPY*, which is another phenotype of the *emp24*Δ condition (Copic et al., 2009). CPY* trafficking to the Golgi is measured by acquisition of an α-1,6-mannose sugar, which represents a quantitative readout of delivery to the Golgi lumen. In WT and *lst1Δ* cells, the misfolded protein fails to reach the Golgi and is rapidly degraded, resulting in low levels of α-1,6-mannose modification. In *emp24*Δ cells, α-1,6-mannose-modified CPY* was increased, as previously reported, whereas deletion of *LST1* reversed this effect (Fig. 6 B). Finally, we confirmed that deletion of Lst1 impacts the anterograde pathway by measuring bulk flow of SP-FLAG-Cp. Compared with the elevated levels of Cp secretion in *emp24Δ* cells, the *emp24Δ lst1*Δ double mutant showed bulk flow rates identical to WT cells (an average reduction of 18% relative to the *emp24Δ* single mutant; Fig. 6 C).

**Figure 7.**
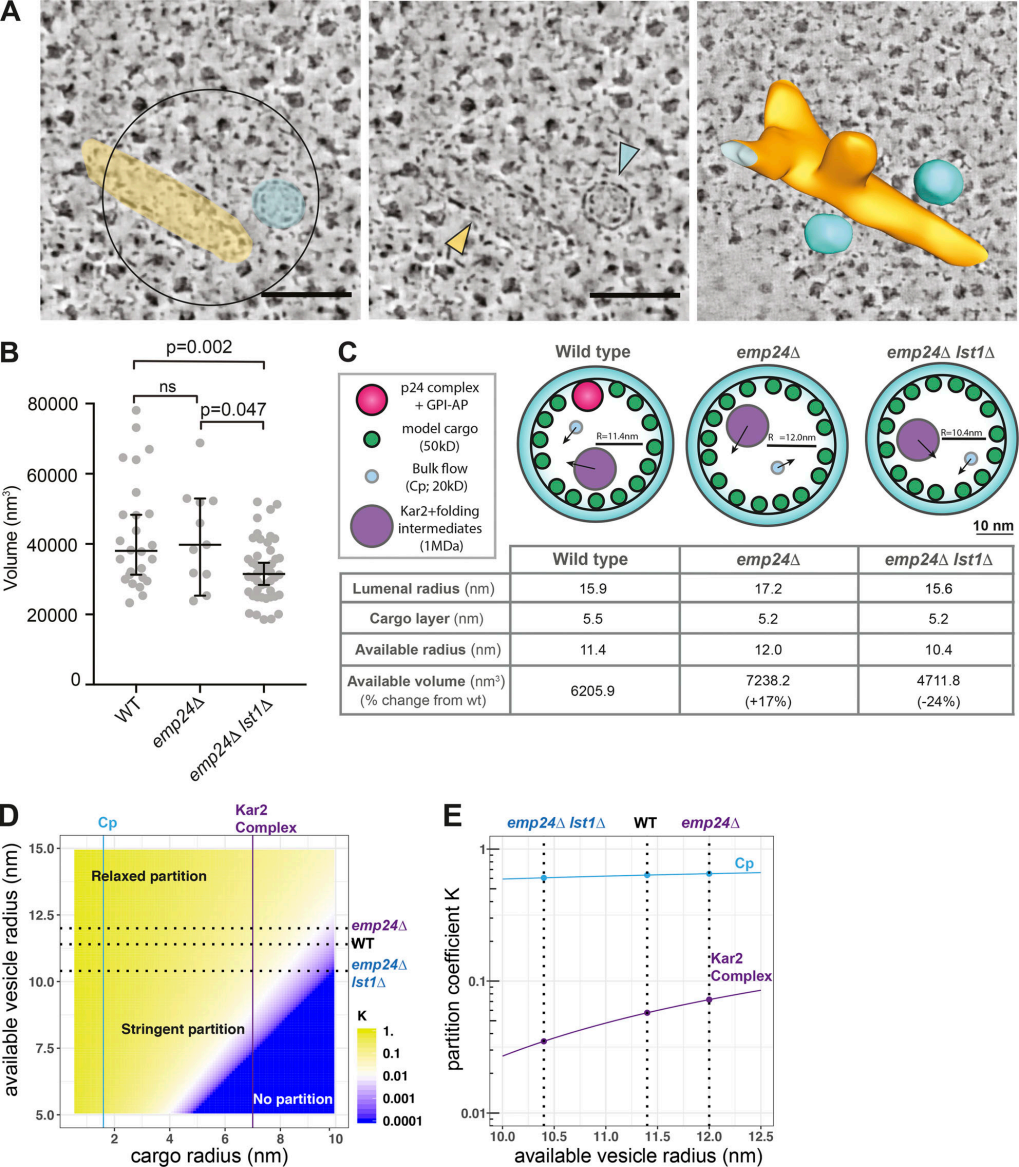
**The volume of COPII vesicles is significantly reduced in *emp24Δ lst1Δ* cells.** **(A)** A Sec16-sfGFP–positive ERES in an *emp24*Δ *lst1*Δ cell. Left panel is a virtual tomographic slice showing a false-colored ER tube (yellow) and a free vesicle (cyan). Central panel shows the same structures highlighted by colored arrowheads. Right panel is a segmentation model of the corresponding 3D membrane ultrastructure showing a bud and two vesicles. Scale bar, 100 nm. **(B)** Plot of the volume (nm^3^) of COPII vesicles in the strains indicated. *n* = 26 for Sec16-sfGFP, *n* = 11 for *emp24Δ*, *n* = 46 for *emp24Δ lst1Δ.* Bars correspond to median value and 95% confidence interval. Statistical test was a one-way ANOVA with Tukey’s correction for multiple comparisons; ns, not significant. **(C)** Model of cargo crowding in vesicles. 2D sections of vesicles drawn to scale illustrating the cargo crowding effects in different strains. The radius of the vesicle lumen and the space occupied by the selected cargo layer (green) in a given vesicle determines the free volume available to bulk flow. Different bulk flow cargoes (cyan and purple circles) will access this space differently based on their size (see Materials and methods for calculation details). **(D)** Heatmap showing changes in K as a function of R_av_ and r_cargo_. Depending on the relative size difference between the available size of vesicle and size of the cargo molecule, the partitioning process could either be less sensitive (relaxed partition) or more sensitive (stringent partition) to the size of a vesicle. Dashed lines mark the vesicle sizes associated with different genetic backgrounds. Solid lines mark the specific cargo, Cp (cyan) and a Kar2/client complex (purple). **(E)** Changes of K for Cp (cyan) and a Kar2/client complex (purple) as a function of vesicle size. Smaller vesicles tend to have more stringent partitioning compared with larger ones, and the size of vesicles impacts partitioning of larger bulk-flux cargoes more significantly than smaller cargoes (see Materials and methods for calculation details). Dashed lines mark the vesicle sizes associated with different genetic backgrounds.

Having demonstrated that deletion of *LST1* indeed restores sorting stringency, we sought to quantify the effect of loss of Lst1 on vesicle size in situ using CLEM. We acquired 37 tomograms at sites of COPII vesicle formation in a *lst1*Δ *emp24*Δ *SEC16-sfGFP* strain (Fig. 7 A). Most of these ERES had more than one adjacent free vesicle, which we segmented to quantify for volume. Vesicles in the *lst1*Δ *emp24*Δ strain had a median volume of 31,493 nm^3^ (Fig. 7 B), corresponding to a volume reduction of 21% compared with *emp24*Δ. The majority of COPII vesicles in the *lst1*Δ *emp24*Δ strain fell below the median vesicle volume of wild-type *SEC16-sfGFP* and *emp24*Δ *SEC16-sfGFP* cells. The smallest vesicles for all strains tested had similar volumes, which highlights that the formation of large COPII vesicles is specifically impaired when Lst1 is deleted.

**Figure S5.**
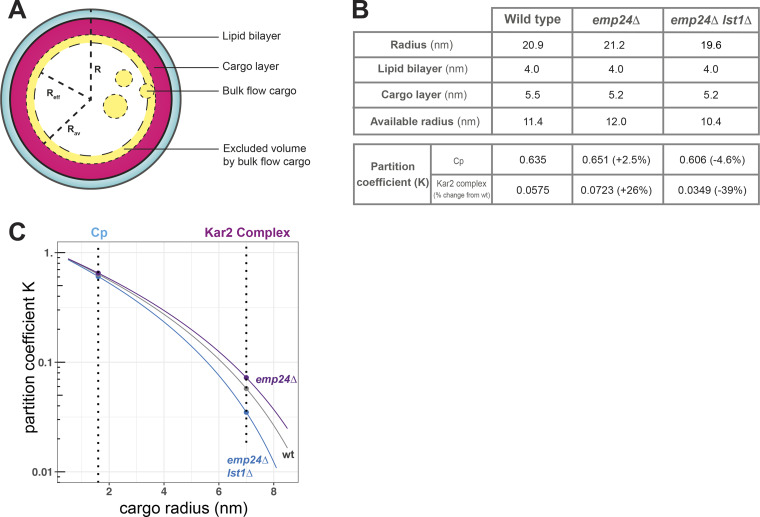
**Partitioning model for cargo occupancy in a COPII vesicle**. **(A)** Model of a COPII vesicle and parameters used in the calculation. The vesicle consists of a lipid bilayer (cyan), a cargo layer (pink), and available space (denoted by short dashed circle). Bulk flow cargoes of different sizes sample different effective volumes (denoted by long dashed circle) during diffusion as a result of the excluded volume effect (denoted by the yellow layer). The radius of effective volume (R_eff_) is determined by the R_av_ and the r_cargo_. **(B)** Parameters and results of calculation. K for smaller cargoes is greater than that of larger cargoes, but the percent change relative to WT is greater for the larger cargo. **(C)** Changes of K as a function of cargo size, dashed lines mark the size of Cp and a Kar2 complex discussed in this work. From the plot it is clear that smaller vesicles tend to have more stringent partition compared with larger ones.

Together our data suggest that cargo occupancy in ER-derived vesicles inversely correlates with the degree of bulk flow, and that the effect of cargo occupancy is to create crowding effects that may serve in part to prevent inappropriate export of ER residents. Since our 3D measurements of vesicle volume in different strains permits a quantitative analysis, we sought to model whether the effects we measured are consistent with theoretical calculations of vesicle size and cargo occupancy (Fig. 7 C and Fig. S5). We reasoned that actively recruited cargo proteins, engaged with Sec24/Lst1, will occupy the volume immediately underlying the membrane and thereby reduce the internal radius of the vesicle (Fig. S5 A). For simplicity, we considered only two classes of cargo proteins, each modeled as spheres: the p24 proteins in complex with a GPI-AP cargo, modeled as a 100 kD complex, and a model “average” cargo of 50 kD (Lodish et al., 2012). Since GPI-APs make up ∼25% of the GFP-secretome (Fig. S3 A), we used a weighted average (25% p24/GPI-AP, 75% model cargo) to calculate the radius occupied by the cargo layer in a WT cell, with only the model cargo contributing to the *emp24Δ* condition (Fig. S5 B and Fig. 7 C). The radius corresponding to the cargo-occupied layer was subtracted from the total lumenal radius to yield an available radius (R_av_) and hence available volume that is accessible to nonselective capture. Comparing the WT condition with that of an *emp24*Δ strain, we calculated an increase of ∼17% in free available volume. This is similar to the degree of increase (∼24%) that we see in bulk flow of Cp in this background (Fig. 4 B). In the *emp24*Δ *lst1*Δ double mutant, the reduction in vesicle size translates to a reduction in available volume of ∼24% compared with WT (Fig. 7 C). From this calculation, one might expect Cp secretion to be reduced in the *emp24Δ lst1Δ* double mutant relative to WT. Instead, we see equivalent bulk flow, suggesting that our model does not capture the full complexity of cargo sorting and bulk flow. We note that Lst1 itself, as a cargo adaptor, has a preference for large cargoes (Miller and Schekman, 2013). Our experimental observations might be explained by a thinner cargo layer in the double mutant, restoring the free available volume back to wild-type levels.

Simple changes in free lumenal volume may account for the changes in Cp secretion in different mutants, but we questioned whether these relatively small differences explain the effects we observe for larger cargo, like Kar2. We therefore sought to model partitioning effects, where the size of a molecule impacts its packaging. Specifically, the radius of a cargo protein determines an excluded volume within the vesicle that restricts cargo access (Fig. S5 A). This effect can be measured by a partitioning coefficient (K), which describes the ratio of concentration of a specific cargo within a confined space (e.g., vesicle) relative to the concentration in the bulk fluid (e.g., ER lumen) at equilibrium. If we assume both cargo and vesicle are spheres, $K={\left(1-\frac{r_{cargo}}{R_{av}}\right)}^{3},$ and can be calculated for each combination of cargo radius (r_cargo_) and R_av_ of a vesicle (Fig. S5 A and Fig. 7 D; see Materials and methods for details). Our model suggests that cargo partitioning can be categorized into three different regimens: (1) relaxed partitioning where R_av _>> r_cargo_, such that cargo capture is largely unaffected by changes in vesicle size; (2) no partitioning where R_av _< r_cargo_, and cargo is thus unable to be packaged at all; and (3) stringent partitioning where R_av_ and r_cargo_ are of similar scales, and cargo partitioning will be sensitive to small changes in vesicle size (Fig. 7 D).

Within the stringent partitioning regimen, several phenomena emerge. First, vesicles of all sizes can discriminate robustly according to cargo size (Fig. 7 E). Thus, small cargoes like Cp are favored by partition and have a relatively high K value. In contrast, K is low for large cargo like Kar2, which is likely engaged with unfolded clients and/or ER cochaperones, and is modeled here as a ∼1 MDa complex (Tatu and Helenius, 1997; Meunier et al., 2002). The very low partitioning coefficient for a large cargo such as a Kar2/client complex suggests that bulk flow capture into a vesicle is unfavorable, and this effect may contribute to robust ER retention. A second feature within the stringent partitioning regimen is that partition of a large cargo is more subject to small changes in vesicle size than a smaller cargo (Fig. 7 E and Fig. S5 C). For example, the partitioning coefficient for the Kar2 complex increases significantly as vesicle size increases, with a 25% change in *emp24Δ* vesicles relative to WT (Fig. 7 E and Fig. S5 B). In contrast, partitioning of Cp is barely affected by changes in vesicle size (2.5% change in K; Fig. S5 B), suggesting simple available volume is a more important factor in its bulk flow traffic. We note that our Kar2 secretion assays do not permit us to quantify the percentage of total Kar2 that is released to the cell surface. In the context of our experimental findings, relatively subtle changes in partitioning of a Kar2/client complex might suffice to saturate a finely tuned retrieval system, resulting in surface detection. Cellular conditions are clearly more complex than a partitioning model captures, but these calculations support our hypothesis that ER leakage results from alterations in the biophysical environment when cargo packaging is altered.

## Discussion

ER export of secretory proteins represents an important quality control checkpoint, whereby folded cargoes are enriched in the nascent vesicle while immature and misfolded proteins, along with ER resident proteins, are excluded. Here, we describe a biophysical mechanism that contributes to such sorting stringency, which derives from the principle that secretory cargoes are not inert passengers, but can confer constraints on vesicle formation. One consequence of cargo enrichment is the requirement for force generation by the vesicle coat, in the absence of which membrane deformation becomes less uniform. A second outcome of cargo packaging into a confined space is that steric pressure derived from molecular crowding can prevent improper capture of nonspecific cargo.

### Coat composition drives vesicle morphology

Our visualization of membrane morphology by CLEM revealed that in the absence of bulky cargo proteins and Sec13, ERES became more pleiomorphic, with individual vesicles generally larger in size. This altered membrane architecture supports the notion that the outer coat scaffold enforces order during vesicle formation. The increase in vesicle size observed in the Sec13-free state is consistent with a reduced ability of Sec31 alone to generate curvature (Copic et al., 2012). However, smaller vesicles of 45–55 nm in diameter were still observed in the absence of Sec13, suggesting Sec31 alone can achieve high curvatures. Whether this smaller population of vesicles has a distinct cargo composition that is more permissive to curvature generation remains to be determined. Traffic of large cargo in mammalian cells is also dependent on Sec13 (Townley et al., 2008), suggesting our findings apply generally to the mechanism of membrane curvature by the COPII coat. Another feature of ERES under Sec31-only conditions was the increased prevalence of multibudded structures still attached to the ER. This may be a consequence of frustrated budding events where Sec31 fails to deform the membrane to a fission point. If this is the case, it suggests a second structural role for Sec13 in ensuring adequate curvature to drive timely vesicle fission.

Our CLEM analysis of *emp24Δ lst1Δ* cells confirmed in vitro observations (Shimoni et al., 2000; Miller et al., 2002) that the composition of the inner coat layer also contributes to vesicle architecture, with the absence of Lst1 yielding a reduction in vesicle volume. The mechanism by which different cargo adaptor isoforms yield vesicles of distinct size remains to be seen. Given the capacity for the inner COPII coat to form oligomeric arrays, it is possible that Lst1 contributes directly to curvature generation to both form a larger structure and provide force to counter the physical effects of its large clients. One corollary of generating large vesicles is the risk of excessive bulk flow if the cargo capacity is not met. Thus, cargo-driven modulation of vesicle size via coat adaptors represents a simple model by which secretion can be tuned to reflect cargo needs. Large cargoes, such as the plasma membrane H^+^-ATPase, Pma1, and GPI-APs, have a preference for Lst1 (Roberg et al., 1999), presumably because of the need for a larger vesicle. By recruiting Lst1 only to ERES that contain these cargoes, the cell might avoid generating larger structures than necessary. Thus, cross-talk between cargo and coat can tailor vesicle size to fit cargo exigency while still maintaining high sorting stringency.

### Vesicle size and cargo occupancy as drivers of quality control

As abundant constituents of COPII vesicles, the p24 proteins (and associated cargoes) may act simply as molecular ballast to occupy space and thus exclude ER residents and minimize bulk flow (Kaiser, 2000). However, the capacity of these proteins to oligomerize, in coordination with the coat, could additionally facilitate the exclusion of noncargo proteins (Ma et al., 2017). The fact that deletion of another abundant cargo, the cell wall mannoprotein Ccw12, similarly caused ER leakage indicates that enforcing sorting stringency is not an exclusive property of p24 proteins, but can be imposed by other bulky cargo. Additional support for a steric model comes from our observation that sorting stringency was restored by an artificial increase in cargo crowding (Fig. 5) or by reduction in vesicle size (Fig. 6). Our partitioning calculations further support the model that retention of ER residents is a biophysical effect that can arise from the limited available volume inside a budding vesicle, combined with active cargo selection by the coat (Ma et al., 2017).

A model for ER retention driven in part by molecular crowding has interesting implications for how cells might adapt to changes in secretory burden. Cargo-driven programming of vesicle size is also likely to occur in mammalian cells. The collagen export receptor, TANGO1, has been proposed to drive coat organization to favor tubule formation around an export-competent collagen fiber (Saito et al., 2009; Raote et al., 2018). However, other modes of collagen export have also been proposed, where local membrane fusion and remodeling might occur (Malhotra and Erlmann, 2015; McCaughey et al., 2019). Whether and how ER resident proteins are excluded from such structures remain to be fully explored. In this context, experiments that abrogate secretion of bulky clients in specialized cell types, for example IgM in plasma cells or mucins in goblet cells, would be informative. Monitoring secretion of ER residents and ERES abundance would reveal how such cells might compensate for loss of cargo crowding effects. To minimize ER leakage, a cell could reduce the number and size of vesicles to ensure maximal cargo packing. Indeed, signaling cascades that modulate vesicle formation according to cargo requirements have been recently described (Subramanian et al., 2019; Centonze et al., 2019), and such pathways may tune ERES activity in the context of cargo abundance. Alternatively, depending on their specific physiology, some cell types might tolerate a degree of constitutive ER leakage that accompanies changes in cargo burden. For example, in professional secretory cells, abundant cargo is packaged at the prevailing ER lumenal concentration (Martínez-Menárguez et al., 1999), and bulk flow leakage may be tolerated, either by enhanced Golgi–ER retrieval or alternative mechanisms. Finally, similar principles may act at other trafficking routes. Fluid phase uptake from the cell surface is well established but may differ under conditions where active cargo capture is enhanced, or vesicle formation becomes limiting. Biophysical effects in clathrin-independent endocytosis are of particular interest, where uptake of proteins can be determined by steric bulk (Bhagatji et al., 2009). Dissecting these different pathways and exploring molecular crowding in vesicles more broadly should shed further light on how cells manage the stringency of protein delivery.

## Materials and methods

### Strains and plasmids

All strains were constructed and grown using standard *Saccharomyces cerevisiae* methods. Strains (Table S1) were made by crossing, sporulation, and dissection of tetrads or by PCR-based integration of auxotrophic or drug-resistance markers. Plasmids used in this study are listed in Table S2. The *pSP-GFP-TM* construct containing the Kar2 signal sequence followed by superfolder GFP and the transmembrane and cytosolic domains of Emp24 (residues 153–203) was purchased as a synthetic construct in pRS316 (GeneScript). The *pSP-GFP-CCTM* plasmid contains the same sequence with the addition of the coiled-coil sequence of Emp24 (residues 129–152) upstream of the transmembrane domain, and was also synthesized commercially (GeneScript). The *pSP-GFP-26xLeu* construct was created from *pSP-GFP-TM*, where the Emp24 transmembrane domain (residues 173–193) was replaced by 26 leucines using Gibson assembly (New England Biolabs). To construct *pSP-FLAG-Cp,* the sequence containing the HA epitope and that of the Semliki Forest virus Cp was amplified from plasmid p626 (a gift from A. Helenius, ETH Zurich, Zurich, Switzerland) and ligated into pRS426. In the resulting plasmid, pRS426-HA-Cp, the HA sequence was replaced by the Ost1 signal peptide followed by the FLAG epitope, using sequential reactions of QuikChange multi site-directed mutagenesis (Agilent).

### CLEM

CLEM was performed as described in Kukulski et al. (2011) with modifications described in Ader and Kukulski (2017). Yeast cells were grown at 25°C in minimal media lacking tryptophan to 0.6–0.8 OD_600_ and pelleted by vacuum filtration on nitro-cellulose discs, then placed on an agar plate to prevent the pellet from drying out. The resulting yeast paste was high-pressure frozen in 200-µm-deep wells of aluminum carriers (Wohlwend) using a HPM100 (Leica Microsystems). Freeze substitution and Lowicryl HM20 (Polysciences, Inc.) resin embedding were done as previously described in Kukulski et al. (2011). 0.03% uranyl acetate in acetone was used for freeze substitution. Samples were shaken on dry ice for the first 2–3 h of freeze substitution. Sections of 300-nm thickness were cut with an Ultra 45° diamond knife (Diatome) on an Ultracut E microtome (Reichert). The sections were floated onto PBS and picked up with 200 mesh carbon-coated copper grids (AGS160, Agar Scientific). Fluorescent TetraSpeck beads (Invitrogen), 50 nm in diameter, were adsorbed onto the grid. Directly after sectioning, grids were mounted for fluorescence microscopy (described below). Prior to electron tomography, 15-nm gold beads (Electron Microscopy Sciences) were adsorbed on the sections, which were then post-stained for 15 min with lead citrate. Scanning transmission EM tomography was done on a TF20 microscope (FEI) with an axial brightfield detector, using a camera length of 200 mm and a 50-mm C2 aperture (Ader and Kukulski, 2017; Hohmann-Marriott et al., 2009). For correlation to fluorescence images, low magnification tilt series at 3.1 nm pixel size were acquired using SerialEM (±55° tilt range, 2° increment, single axis acquisition; Mastronarde, 2005). Higher magnification tomograms were acquired with dual axis tilt series ±60° with 1° increment and at 1.1 nm pixel size (Mastronarde, 1997). All tomographic reconstructions were done in IMOD (Kremer et al., 1996), and fiducial-based correlation was done using MATLAB-based scripts described in Kukulski et al. (2011).

### Segmentation analysis

The 3D membrane morphologies of correlated ERES shown in the figure panels were segmented by manual tracing, simplification, and smoothening of the resulting surfaces using Amira (Thermo Fisher Scientific). Thereby generated models are for illustration purposes only.

Vesicles diameter and volume quantifications were obtained with FIJI using the plugin LimeSeg (Machado et al., 2019) as follows: the outer contour of a vesicle was selected to the region of interest (ROI) using the “point tool” and “segmented line” tool moving in z through the tomographic slices, adding to the ROI the lowest plane of the vesicle with the point tool, then clicking contours with the segmented line tool every five virtual slices and finally closing the volume selecting the top plane of the vesicle with the point tool. LimeSeg Skeleton Segmentation tool settings were adjusted to recognize and segment the outer surface of the vesicle (D_0: 4, F_pressure: 0, Z_scale: 1, Range_in_DO_units: 1, NumberOfIntegrationStep: −1, RealXYPixelSize: 1). After running the segmentation, the correct distribution of surfels over the outer contour of the vesicle was assessed by eye. The LimeSeg segmentation tool provides the list of vertices of the mesh. The centroid of this point cloud gives an estimate the center of the segmented vesicle. The maximum radius is then computed as the maximum distances to this center taking into account the 1.1 × 1.1 × 1.1 nm voxel size.

### Secretion assays

To examine Kar2 secretion on plates, serial dilutions of logarithmic phase cultures were spotted onto yeast extract peptone dextrose (YPD) plates and incubated at 30°C for 5 h, at which point colonies were overlaid with nitrocellulose filters and incubated for a further 1 h. Nitrocellulose filters were washed, blocked, and incubated with α-Kar2 polyclonal sera (provided by R. Schekman, University of California, Berkeley, Berkeley, CA). Secreted Kar2 was detected with HRP-conjugated goat-anti-rabbit antibodies followed by ECL detection (Pierce). Where protein secretion was monitored from liquid cultures, stationary phase cultures were back diluted into fresh media and grown for 5 h. Two OD_600_ units of logarithmic phase cells were collected by centrifugation at 14,000 *g* for 5 min and 1.5 ml of the supernatant fluid collected. Proteins in the media fraction were precipitated by adding 0.15 ml of 100% TCA (Sigma Chemical) and incubated on ice for 30 min. Precipitated proteins were collected by centrifugation, washed with acetone, dried, and resuspended in 30 µl of SDS-PAGE sample buffer supplemented with 50 mM Tris, pH 9.4. One fifth of the sample was resolved by SDS-PAGE for Kar2 immunoblots and one third for SP-FLAG-Cp or SP-GFP immunoblots. Cell pellets from the two OD_600_ units were lysed to obtain whole cell preparations. One tenth was analyzed by immunoblot.

### FLIP assays

FLIP was used to measure mobility of Kar2-sfGFP. FLIP was selected as the method rather than fluorescence recovery after photobleaching (FRAP) because the small size of yeast cells makes quantification of such experiments difficult. Identical ROIs containing a similar fraction of the ER were continuously photobleached in roughly similar sized cells. Imaging was performed in cells grown to mid-log phase at 30°C in minimal media lacking tryptophan. Images were taken on an Andor Revolution Spinning Disk microscope with a 40×/1.3 NA oil immersion objective and an electron multiplying charge-coupled device (EMCCD) camera. Images were collected using the Andor iQ3 software. A small region of interest was repeatedly photobleached, and the fluorescence intensity of the whole cell was measured for a loss of signal, representing protein that had diffused into the bleaching area. Fluorescence intensity was measured using Fiji, and statistical analysis was performed with Prism 7.0 (GraphPad Software).

### GFP imaging

Emp24-GFP and Erd2-GFP imaging was performed in cells grown to mid-log phase at 30°C in minimal media lacking tryptophan. Images were taken on an Andor Revolution Spinning Disk microscope with a 40×/1.3 NA oil immersion objective and an EMCCD camera. For imaging of Sec16-sfGFP and Sec24-sfGFP, cells were grown at 25°C in minimal media lacking tryptophan. Images were taken on a Nikon Ti2 with a 100×/1.49 NA Oil (TIRF) objective and a scientific complementary metal–oxide–semiconductor (sCMOS) camera. The same imaging methodology was used for the imaging of EM grids with section of resin-embedded cells.

### Western blot

Total protein extracts prepared by alkaline lysis of exponentially growing yeast were separated by SDS-PAGE. Proteins were detected with corresponding antibody, and chemiluminescence was visualized according to the manufacturer's instructions (ECL Advanced; GE Healthcare).

### Pulse chase analysis of protein secretion and trafficking

Pulse chase experiments were used to monitor secretion of the bulk flow marker SP-FLAG-Cp and intracellular transport of Gas1, CPY, and the misfolded form of CPY, CPY*. These experiments were performed as previously described (Pagant et al., 2007). Briefly, strains were grown to mid-log phase at 30°C, starved for 15 min, and labeled for 5 min with 1 µl per OD of cells of EXPRESS ^35^S Protein Labeling Mix (PerkinElmer) for 5 or 10 min. The label was chased with excess rich media and two OD aliquots of cells harvested at different times. Cells were lysed in detergent, and the protein of interest was immunoprecipitated from cell lysates, and cell media (when measuring SP-FLAG-Cp secretion) were separated by SDS-PAGE and detected by phosphorimaging using a Typhoon scanner (GE Healthcare). The protein bands were quantified using Fiji, and the percentage of the mature or secreted band in each sample was plotted with Prism 7.0 (GraphPad Software).

### Vesicle volume model calculations

Our goal was to estimate the cargo occupancy of a vesicle and thereby determine how cargo enrichment and vesicle size might influence cargo sorting stringency. Using our quantitative segmentation analysis, we obtained the median vesicle volume for WT, *emp24*Δ, and *emp24*Δ *lst1*Δ strains. For simplicity, we assumed these segmented volumes to be spheres. To obtain a value for the lumenal volume, we first calculated the radius of the vesicle from the median volume and subtracted 4 nm corresponding to the lipid bilayer. We then subtracted a cargo-occupied layer that we approximated as a mixture of p24/GPI-AP cargo (25%) and “average” secretory cargo (75%, termed model cargo). We treated these cargoes as spheres and used a weighted average of their diameters to calculate the thickness of this cargo layer. The diameters of specific cargoes were estimated using the online tool Protein Size (http://www.calctool.org/CALC/prof/bio/protein_size) based on molecular weight (50 kD for model cargo, 100 kD for p24/GPI-AP). Subtracting the cargo-occupied layer from the lumenal radius described above, we obtained the $R_{av}$ inside the vesicle that was then used to calculate the available lumenal volume ($V_{av}$) or available space inside the vesicle. The effective volume that the centroid of a cargo can sample ($V_{eff}$) is calculated by $V_{eff}=\frac{4}{3}\pi \;R_{eff}^{3},$ where $R_{eff}=R_{av}-r_{cargo},$ and $r_{cargo}$ is the radius of the specific cargo based on a spherical assumption. Dimensionless factor partition coefficient, K, of a specific cargo in a specific size of vesicle, is defined as $K=\frac{V_{eff}}{V_{av}}$ and further reduced to $K={\left(1-\frac{r_{cargo}}{R_{av}}\right)}^{3}.$ For specific cases, the radius of cargo for Cp was calculated by WinHYDROPRO (Ortega et al., 2011) using the crystal structure of the protease domain of Semliki Forest virus capsid protein (PDB accession no. 1VCP), and the radius of Kar2/client complex is estimated in the same way using Protein Size as described above, assuming the molecular weight of the complex is 1 MDa. The heatmaps and graphs associated with the model were plotted using R.

### Online supplemental material

Four supplemental figures include additional data. Fig. S1 provides an additional example of a correlated tomogram and shows the phenotypes (growth and ERES) associated with different mutants. Fig. S2 shows an additional example of a correlated tomogram in the *emp24Δ sec13Δ* mutant. Fig. S3 provides supporting information about Ccw12 and its mutant phenotypes. Fig. S4 shows a characterization of the model cargo proteins described in Fig. 5. Table S1 and Table S2 describe the yeast strains and plasmids used in this study, respectively.

## Supplementary Material

Table S1 lists yeast strains.

Table S2 lists plasmids.
