# Supplementary material for: Cargo crowding contributes to sorting stringency in COPII vesicles
Source: J Cell Biol. 2020 May 13;219(7):e201806038. doi: 10.1083/jcb.201806038 (PMC7300426; doi:10.1083/jcb.201806038)
Supplement: Table S2 — lists plasmids. [file JCB_201806038_TableS2.docx]

**Table S2: Plasmids**

| **Plasmid** | **Description** | **Source** |
| --- | --- | --- |
| *pGAL-CPY*-HA* | pTS210-GAL1-CPY*-HA | Kawaguchi et al., 2010 |
| *pGAL-CPY*-Δ1-HA* | pTS210-GAL1-CPY*-D1-HA | Kawaguchi et al., 2010 |
| *pSP-GFP-CCTM* | pRS316-*GAL1-SP-GFP-CoiledCoil-TMD- Emp24 cytosolic domain* | GeneScript (This study) |
| *pSP-GFP-TM* | pRS316-*GAL1-SP-GFP-TMD- Emp24 cytosolic domain* | GeneScript (This study) |
| *pSP-GFP-26xLeu* | pRS316-*GAL1-SP-GFP-26xLeu-Emp24 cytosolic domain* | This study |
| *pSP-FLAG-Cp* | p426-*GAL1-SP-FLAG-Capsid protease (Cp) domain* | This study |
| *pER-GFP* | pRS415-ss-sfGFP-Myc | Snapp et al., 2006 |
| *pEmp24-sfGFP* | YCplac111-*EMP24-sfGFP* | D'Arcangelo et al., 2015 |
| *pLST1* | pRS313-*LST1* | D'Arcangelo et al., 2015 |
| *pLST1-B* | pRS313-*lst1*−b (K543M,R545M) | D'Arcangelo et al., 2015 |
